# Which Threats to Global Health Pose a Problem for Turkey’s Health?

**DOI:** 10.4274/balkanmedj.galenos.2019.2019.3.001

**Published:** 2019-05-10

**Authors:** Burcu Tokuç

**Affiliations:** 1 Deputy Editor, Balkan Medical Journal Department of Public Health, Trakya University School of Medicine, Edirne, Turkey

The world today faces multiple health challenges. The World Health Organization (WHO) recently published a list of ten threats to global health in the year 2019. These include outbreaks of vaccine-preventable diseases, increased reports of drug-resistant pathogens, increased rates of obesity and physical inactivity, health effects of climate change and environmental pollution, and multiple humanitarian crises (1). What do these problems mean to Turkey?

## Air pollution and climate change

The WHO considers air pollution as the greatest environmental risk to health in 2019. Nine out of ten people breathe polluted air every day (1). Every year 7 million people die prematurely from diseases, such as cancer, stroke, and heart and lung disease due to microscopic pollutants in the air.

According to the limits set by the WHO, 80 out of 81 provinces of Turkey are exposed to polluted air; furthermore, based on the national air quality limits, 67% of the cities have poor air quality (2). An estimated 30,000 premature deaths occur every year due to air pollution (2). Another glaring indication of the magnitude of this problem is the WHO 2018 data that reveals the incidence of premature deaths due to air pollution in Turkey (46.6 deaths per 100,000 individuals) to be 1.5 times the WHO European region average (3).

Climate change, which affects public health in many different ways, is estimated to lead to 250,000 additional deaths annually from malnutrition, malaria, diarrhea, and heat stress (1). The 2011 National Action Plan on Climate Change revealed that the average annual temperature is estimated to rise by 2.5 °C-4 °C in the next few years in Turkey (4). Despite such foreboding data, Turkey is yet to establish effective environmental protection policies and/or take adequate environmental protection measures.

## Non-communicable diseases

Non-communicable diseases, such as diabetes, cancer, and heart disease, are collectively responsible for over 70% of deaths worldwide, an estimated 41 million people. This covers 15 million premature deaths in people aged 30-69 years. Five major risk factors contribute to the increase in the incidence of these diseases: tobacco use; physical inactivity; excessive intake of alcohol; unhealthy diets; and air pollution.

According to the latest data released by the Ministry of Health (5), the greatest increase in disease burden in 15 years was observed in Alzheimer’s disease (68.4% increase) and in stroke (57.4% increase), while the most significant reduction was observed in lower respiratory tract infections (63.7% decrease). Non-communicable diseases contribute to 87.5% of deaths in Turkey (6). The probability of premature death due to four non-communicable diseases is likely one sixth (16.8%) for an individual in Turkey (6).

A 2017 study of the prevalence of risk factors for non-communicable diseases in Turkey reported tobacco use in 31.6% of individuals. Additionally, 64.4% of individuals in the study group were overweight [body mass index (BMI) ≥25 kg/m^2^], 28.8% were obese (BMI ≥30 kg/m^2^), and 43.6% had insufficient physical activity with median time of 30 min per day spent performing physical activity (6). Most early deaths from non-communicable diseases can be largely prevented by making health systems more equitable and responsive to the healthcare needs of these patients, and by collaborating with non-health sectors.

To carry out these commitments, the World Health Assembly (that Turkey is a member of) approved the 2013-2020 Action Plan for Global Non-Communicable Disease Prevention and Control in May 2013. The goal “To reduce and surveillance of the incidence of non-communicable diseases and their risk factors” has received wide coverage in the Republic of Turkey Ministry of Health Strategic Plan and Action Plan 2013-2017, and some strategies have been identified (7).

The strategies for this goal are as follows: to raise awareness of non-communicable diseases and risk factors; to establish a surveillance system for monitoring and management of non-communicable diseases; and to strengthen the prevention and control programs for non-communicable diseases. However, as of 2019, these strategies have not achieved the desired success in reducing non-communicable diseases in Turkey.

## Antimicrobial resistance

Antimicrobial resistance is one of the most fundamental issues of the global agenda in recent years due to its public health impact and economic cost. In terms of antibiotic use, Turkey ranks first among the Organization for Economic Co-operation and Development countries and second in antimicrobial resistance (8).

The misuse of antibiotics, lack of regulation of livestock, lack of information among health workers, and insufficient political arrangements are the problems faced by Turkey that need effective solutions. One of the priorities in this context is to increase data collection and monitoring mechanisms for all agricultural and health practices. Programs for the reduction of antibiotic consumption have been established by the Ministry of Health in all relevant institutions and organizations in Turkey in recent years, and new policies are put into practice. Significant progress and increased awareness has been created such that antibiotics are no longer sold without prescription in Turkey. Moreover, surveillance studies on the use of antibiotics in both humans and animal husbandry are on the agenda (9).

## Vaccine hesitancy

Unfortunately, since 2013, anti-vaccine groups that confuse people by spreading incorrect information are rapidly increasing in Turkey. Parents are coming together through a platform called “I don’t have to vaccine my child”.

Ensuring high immunization coverage and expanding vaccine access to those who are being missed are crucial parts of universal health coverage. In Turkey, the number of families who refused to have their children vaccinated increased from 11,000 in 2016 to 23,000 in 2018. If vaccination is interrupted, up to 14,000 children may lose their lives each year due to vaccine-preventable diseases leading to an economic loss of 20 billion euros (10).

Vaccination services are a public responsibility. Therefore, in the light of scientific data, the public should be informed about vaccine-preventable diseases, legal arrangements should be made for the protection of the people at risk, and educational tools to disprove anti-vaccine theses should be developed. Turkey is yet to take effective steps in this regard. It should be noted that the government’s inability to enact laws on this subject can be considered as a criminal offense against the positive duty obligation. The authorities should follow a clear and consistent approach.

## Fragile and vulnerable settings

A large fraction (22%) of the world’s population lives in countries with prolonged humanitarian crises, grappling with the challenges of drought, famine, conflict, and population displacement. Turkey has a significant refugee problem because of these conditions at the country’s borders.

The refugee population that escaped the war in Syria exceeds 3.6 million, of which 380,000 are babies born in Turkey (11).

For preventing the problems faced by more than 3 million Syrians in the places where they currently live and to ensure that health services are easily accessible to them, Migrant Health Units have been established wherein primary health care services are provided. Currently, 152 Migrant Health Centers are in service in Turkey (11).

Although a large number of Migrant Health Centers have been set up, they merely strengthen health systems to combat war and hunger and constitute a temporary solution without fixing the main problem. Unfortunately, the main problem can only be solved by the initiatives of the international community.

## Weak primary health care

The primary health care (PHC) system meets a majority of an individual’s health care needs throughout their life. A healthcare system with a strong PHC always provides better and more efficient healthcare and quality care. Health systems need strong PHC at their core if they are to achieve universal health coverage and health-related sustainable development goals (12).

Innovations in health care, arrangements in primary care and family medicine system, and effective and accountable health management are the key steps toward our goal for strengthening PHC services.

## Human immunodeficiency virus

According to the Turkish Ministry of Health statistics, since the first human immunodeficiency virus/AIDS case in 1985 in Turkey 20,293 people have been diagnosed as human immunodeficiency virus positive and the number of human immunodeficiency virus positive patients has increased by 465% over the past 10 years (13). Therefore, Turkey has experienced the most increase in the number of human immunodeficiency virus positive patients in the world. With the support of the WHO, Turkey introduced self-testing to provide information to maximum human immunodeficiency virus positive patients about their status for treatment or preventive measures.

## Global influenza pandemic

More influenza pandemic is expected; however, experts do not know where, when, and how severe will it be. In case of a global outbreak, the health system should be strengthened and prepared for emergencies.

## Ebola and dengue

Ebola and several other hemorrhagic fevers, including Zika, Nipah, Middle East respiratory syndrome coronavirus, severe acute respiratory syndrome, disease X, and dengue, that could cause serious epidemics have not yet been health threats for Turkey.

The above-mentioned list is an indication of changing health challenges, and that non-communicable diseases and environmental threats pose just as great a risk for the future. While talking about threats to health, there is a need to focus on systemic problems that threaten our healthy living conditions, while emphasizing health services. The underlying causes of non-communicable diseases are systemic and environmental problems that cannot be solved by health services alone. Therefore, emphasizing the effects of environmental pollution in our country (where the environmental struggle is becoming increasingly difficult), advocating for the health of the public and not the interests of the market, and sustaining this struggle in an organized manner within local, national, and international organizations is very crucial.

The WHO published a list of ten health threats that will require more attention in 2019. To address these issues, the WHO is beginning a 5-year strategic plan, the 13th General Program of Work. The plan has a triple billion target to ensure that 1 billion more people benefit from access to health care, 1 billion more people are protected from health emergencies, and 1 billion more people experience better health and well-being (1). Turkey should aim to achieve these goals at the national level.

## References

[1] Ten threats to global health in 2019 (cited 2019 January 31).

[2] Clean Air Platform. Air Pollution in Turkey: Black Report (cited 2019 February 1).

[3] World health Statistics 2018 (cited 2019 February 1).

[4] Küresel İklim Değişikliği ve Türkiye (cited 2019 February 1).

[5] Turkish Ministry of Health (2018.). Turkey Statistics Yearbook-2017. Ankara.

[6] Üner S, Balcılar M, Ergüder T (2018.). Türkiye Hanehalkı Sağlık Araştırması: Bulaşıcı Olmayan Hastalıkların Risk Faktörleri Prevalansı 2017 (STEPS). Dünya Sağlık Örgütü Türkiye Ofisi, Ankara.

[7] Republic of Turkey Ministry of Health Strategic Plan and Action Plan 2013-2017 (cited 2019 February 2).

[8] Antimicrobial resistance (cited 2019 February 2).

[9] TEPAV (2017.). Türkiye’de Antimikrobiyal Direnç: Ekonomik Değerlendirme ve Öneriler. Ankara,.

[10] The horrific trend of the anti-vaccine movement in Turkey (cited 2018 February 8).

[11] Gültaç AS, Yalçın Balçık P (2018). Türkiye’de Suriyeli Sığınmacılara Yönelik Sağlık Politikaları. Sakarya Tıp Dergisi.

[12] Primary Health Care (cited 2019 February 19).

[13] Number of HIV patients in Turkey up fourfold in last 10 years (cited 2019 February 1).

